# Comparison of the Adsorption and Desorption Dynamics of Biological Molecules on Alginate Hydrogel Microcapsules—The Case of Sugars, Polyphenols, and Proteins

**DOI:** 10.3390/gels10030201

**Published:** 2024-03-16

**Authors:** Maja Benković, Izvorka Laljak, Davor Valinger, Tamara Jurina, Tea Sokač Cvetnić, Jasenka Gajdoš Kljusurić, Ana Jurinjak Tušek

**Affiliations:** Faculty of Food Technology and Biotechnology, University of Zagreb, Pierottijeva 6, 10000 Zagreb, Croatia; i.laljak1@gmail.com (I.L.); davor.valinger@pbf.unizg.hr (D.V.); tamara.jurina@pbf.unizg.hr (T.J.); tsokac@pbf.hr (T.S.C.); jasenka.gajdos@pbf.unizg.hr (J.G.K.); ana.tusek.jurinjak@pbf.unizg.hr (A.J.T.)

**Keywords:** alginate hydrogel microcapsules, gallic acid, glucose, bovine serum albumin, adsorption and desorption kinetics

## Abstract

The aim of this work was to analyze and compare the adsorption and desorption processes of carbohydrates (glucose as a model molecule), polyphenols (gallic acid as a model molecule), and proteins (bovine serum albumin, BSA as a model molecule) on alginate microcapsules. The adsorption and desorption processes were described by mathematical models (pseudo-first-order, pseudo-second-order, and Weber–Morris intraparticle diffusion model for adsorption, and first-order, Korsmeyer–Peppas, and the Higuchi model for desorption) in order to determine the dominant mechanisms responsible for both processes. By comparing the values of adsorption rate (*k*_2_) and initial adsorption rate (*h*_0_) based on the pseudo-first-order model, the lowest values were recorded for BSA (*k*_1_ = 0.124 ± 0.030 min^−1^), followed by glucose (*k*_1_ = 0.203 ± 0.041 min^−1^), while the model-obtained values for gallic acid were not considered significant at *p* < 0.05. For glucose and gallic acid, the limiting step of the adsorption process is the chemical sorption of substances, and the rate of adsorption does not depend on the adsorbate concentration, but depends on the capacity of the hydrogel adsorbent. Based on the desorption rates determined by the Korsmeyer–Peppas model (*k*), the highest values were recorded for gallic acid (*k* = 3.66236 ± 0.20776 g beads/mg gallic acid per min), followed by glucose (*k* = 2.55760 ± 0.16960 g beads/mg glucose per min) and BSA (*k* = 0.78881 ± 0.11872 g beads/mg BSA per min). The desorption process from alginate hydrogel microcapsules is characterized by the pseudo Fickian diffusion mechanism.

## 1. Introduction

Natural bioactive molecules, proteins, and sugars represent a backbone of food production, mainly due to their ability of texture and taste modification, but also as bearers of functional properties with diverse benefits to human health. However, a constant challenge for their use in food products is their easy degradation, especially during thermal processing. Their stability can be improved by microencapsulation methods, where an active substance sensitive to external conditions is embedded inside a protective polymer material. Microencapsulation is a widespread method for preserving and stabilizing functional compounds for food, pharmaceutical, and cosmetic applications [1].

Alginate, a natural anionic polymer obtained mainly from brown algae, has found application in various fields thanks to its biocompatibility, biodegradability, availability, chemical stability, and the ability to form stable hydrogels. Furthermore, it has a GRAS status and can therefore be used in a variety of foods, including foods for infants and young children with special medical purposes [2], meat, seafood, fruit, vegetables, pasta, noodles [3]; encapsulation of various bioactives and probiotics, which can then be added to food products such as ice cream, yoghurt, mayonnaise, protein isolates, etc. [4]. In microencapsulation processes with alginate as a polymer, gel formation occurs immediately after contact with the receiving ion solution, and the process itself is therefore simple, carried out in mild and nontoxic conditions, and does not require complicated devices, which is why alginate is widely used for microencapsulation methods [5]. In addition to alginate, other biopolymers such as chitosan, gelatin, or cellulose can be used [6,7] and their choice depends on their adsorption and desorption properties for a given molecule, as well as their behavior during gelation.

When designing microencapsulated compounds that are adequate for use in food products or drug delivery, in addition to their influence on structural and sensory properties, it is important to make an in-depth analysis of their adsorption and desorption or release dynamics from the hydrogels used for their formation, and to experimentally determine the coefficients that characterize the transport of sorbate inside the sorbent [8]. Also, the use of kinetic models gives an insight into the efficiency of the hydrogel adsorbent, the rate of removal of the adsorbate from the solution, and the probable thermodynamic profile of the hydrogel system [9].

To understand the characteristics of the hydrogel adsorption process, pseudo-first-order, pseudo-second-order, and Elovich kinetics models are predominantly used, while the Weber–Morris model was designed to describe the mechanism of intraparticle diffusion of the adsorbent [10,11]. Of the many kinetic models developed for the description of the hydrogel desorption processes, some of the most often used are zero-order kinetics model, first-order kinetics model, Korsmeyer–Peppas, Higuchi, Hixson–Crowell, Baker–Lonsdale, Weibull, Hopfenberg, and Gompertz [12].

Up until now, many studies of the process of adsorption and desorption of active substances on microcapsules have been conducted. Natural origin gels were used in many studies which emphasize their functionality as efficient pollutant removers from wastewater [13,14,15], tissue engineering applications [16], and food packaging [17]. Geetha et al. [18] investigated the potential of alginate nanoparticles for the removal of dye (Malachite green) from water using a batch adsorption technique. The effects of pH, initial dye concentration, contact time, temperature, and adsorbent dose on the adsorption rate were investigated. Nochos et al. [19] investigated the kinetics of protein release from alginate/hydroxypropyl methyl cellulose (HPMC) hydrogel microcapsules of different composition. Bušić et al. [20] examined the influence of alginate hydrogels combined with different fillers (cocoa and carob) on the success of encapsulation and desorption of phenolic compounds from dandelion. Benković et al. [21] analyzed the adsorption and desorption processes of bioactive compounds from plants on alginate microcapsules. In addition to the above mentioned, a factor that plays an important role in the adsorption and release is the type of molecule which is being adsorbed or released from the matrix, their structure, and molecular weight. The influence of the chemical structure of the adsorbate and the bonds forming between alginate and biological molecules has been studied in the past: the basic interactions between glucose and alginate are hydrogen bonds [22], and the same is valid for gallic acid [23]. Proteins, on the other hand, can bind to alginate via electrostatic protein–polysaccharide bonds, whereby the oppositely charged amino acids on BSA are bound to the anionic polysaccharide molecules in the alginate [24]. Also, although there is documented research on the effect of the molecular weight and the glucuronic/mannuronic acid ratio of the alginate used to form the hydrogel on the adsorption processes of alginate as adsorbent [25,26,27], to the best of our knowledge, there is little data available on the influence of the type of the most commonly occurring biological molecules and their molecular weight on the adsorption/desorption kinetics. Due to the above mentioned, this paper aims to analyze and compare the adsorption and desorption processes of three types of the most commonly used biomolecules from alginate hydrogel microcapsules: carbohydrates (glucose as a model molecule), polyphenols (gallic acid as a model molecule), and proteins (BSA—bovine serum albumin as a model molecule). Adsorption and desorption processes are then described by suitable mathematical models, and kinetic parameters are estimated and compared to determine the influence of the type of molecule and molecular weight on the aforementioned processes.

## 2. Results and Discussion

### 2.1. Adsorption Experiments

**Figure 1:**
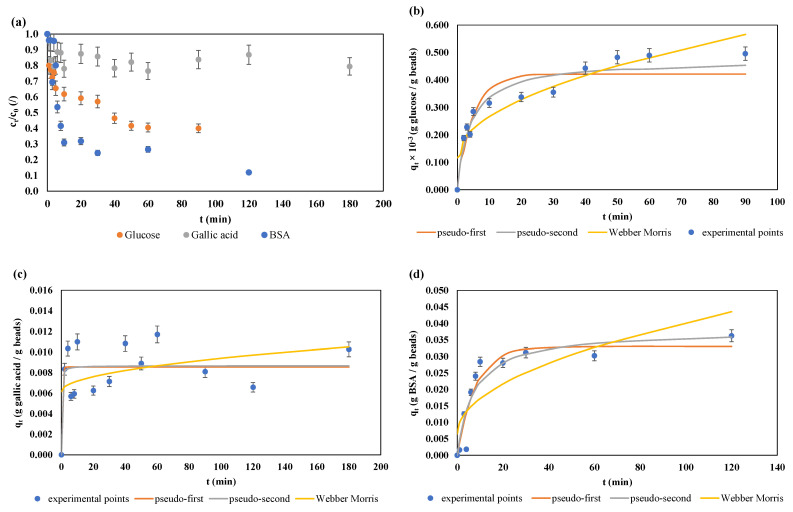
(**a**) Changes in the relative (c_t_/c_0_) concentrations of the supernatant solutions during adsorption and the concentrations of the adsorbate in the alginate hydrogel beads (q_t_) during adsorption (experimental + model fitted data): (**b**) glucose containing alginate hydrogel beads; (**c**) gallic acid—alginate hydrogel beads; and (**d**) BSA—alginate hydrogel beads.

In this work, adsorption and desorption dynamics of three biological molecules which are often used in encapsulation processes were compared: glucose, gallic acid, and BSA protein. Results of the adsorption experiment, seen as the changes in relative concentrations of the given biological molecule in the supernatant and in the alginate hydrogel beads, are shown in Figure 1.

Due to easier comparison of the adsorption processes of glucose, gallic acid, and BSA on alginate hydrogel beads, results for the solutions containing the adsorbate are shown as relative concentrations, calculated as ratios of a concentration in a given time t, and the initial concentration of the adsorbate solution. As shown in Figure 1a, the relative concentration of the glucose solution showed a rapid drop that lasted until t = 5 min of the adsorption process. After that, a decrease in concentration was slower, and after t = 50 min, no significant change in concentration was detected, indicating that the process entered its equilibrium phase. In general, a total of 60% of glucose present in the initial adsorbate solution was adsorbed, of which 35% was in the first five minutes, an additional 23% between t = 5 min and t = 50 min, and only 2% during the remaining 40 min of the process. As seen in Figure 1b, the concentration of glucose in the alginate hydrogel beads rose from 0 to approximately 0.3 mg/g beads in the first few minutes of the adsorption process, and the final concentration of glucose in the beads was 0.495 mg/g beads. One of the possible reasons that only 60% of the total glucose present in the solution was adsorbed can be explained by the low initial concentration of the glucose solution, which limited the contacts of glucose molecules with alginate. Namely, in the work of Tanaka et al. [28], different initial concentrations of glucose solution for adsorption on Ca–alginate gel beads were analyzed and it was concluded that, although glucose diffused freely into 2% alginate beads, the adsorbate solution concentration had a significant influence on the process: at too-high concentrations of glucose solution, diffusion is slowed down, and lower concentrations were recommended as optimal (100–200 mg/mL). However, at lower concentrations, adsorption on Ca–alginate gel beads was hindered by the absence of glucose molecules, which can diffuse into the bead. In more recent studies, Mehmetoğlu and Hacimusalar [29] concluded that the diffusivity of glucose is also influenced by the concentration of the alginate solution and temperature at which the adsorption is performed, while Venâncio and Teixeira [30] concluded that carbohydrates with higher molecular weight demonstrate lower diffusivity. Furthermore, glucose diffusivity is also affected by crosslinking density, polymer concentration, and chemical structure of the adsorbent gel [31]. In addition, discrepancy between the experimental data and the model data is visible in Figure 1b, especially in the part of the adsorption process at t = 20 min and t = 30 min, indicating a lower adsorption rate than expected. This could be due to the existence of a saturation phase where glucose molecules completely cover the bead surface and, until some molecules diffuse into the bead interior, no new molecules can be bound. However, this claim has to be corroborated by future studies. Also, another explanation could be connected to the regions of the adsorption process described by Kalam and coworkers: the adsorption takes place in four regions (phases), where the declining phases (regions 3 and 4) indicate neutralization of the surface and micelle formation, which, as a consequence, slows and halts the adsorption [32]. 

The measured concentrations of gallic acid in the supernatant (Figure 1a) also showed a decreasing concentration trend, but with numerous deviations from the observed trend. The same was observed for the concentrations of gallic acid adsorbed on the gel beads, with the final concentration of gallic acid in the alginate hydrogel beads being 0.010 g/g beads (Figure 1c). In the first four minutes, the concentration consistently decreased, then from t = 6 min to t = 60 min, several alternating jumps and falls were measured, then an increase until t = 120 min, and a drop at t = 180 min. In total, about 20% of gallic acid was adsorbed, which was achieved by the fourth minute, after which the adsorption effect varied and ranged between 11% (t = 6 min) and 23.5% (t = 60 min). Therefore, it can be concluded that the adsorption peaked at t = 60 min. These results indicate low binding of gallic acid to the alginate gel beads, which would suggest that, when encapsulating gallic acid, the alginate should be dissolved directly in the gallic acid solution, which can result in high encapsulation efficiencies. Higher encapsulation efficiency of gallic acid in alginate hydrogels, when the polymer is directly diluted in the acid solutions, ranging from 64.11 to 66.30% was demonstrated in a study by Essifi et al. [33], while numerous different studies demonstrated high encapsulation efficiencies of other polyphenols in polymer gel matrices [20,21,34]. On the other hand, in another study by Essifi et al. [35], lower encapsulation efficiencies were obtained (13.92 to 39.66%), which were explained by the influence of alginate concentration: the observed increase in encapsulation efficiency with the decrease in sodium alginate concentration indicated the formation of a hydrogel network structure without homogeneous distribution of pores and their size, which can retard the diffusion of gallic acid towards the external medium (collection solution) during the gelling process. Another important difference during the adsorption processes in this study is the pH value. Namely, while the glucose and the BSA solutions had pH values ranging from 7.24 to 8.20, the gallic acid solution had a markedly lower pH of 3.26. At higher pH values, chain expansion of the ionic carboxylate groups occurs, which leads to higher swelling ratio of alginate, while at lower pH values, alginate will shrink and the beads will have a more irregular, oblate shape [36]. Also, the mechanical strength of the beads decreases at lower pH values due to decrease in the dissociation degree and intermolecular entanglement [24]. In this case, the mechanical strength of the beads submerged in a low pH solution could have resulted in low protective properties towards gallic acid and, thus, the lack of a clearly visible adsorption trend. 

The relative concentration changes of the BSA solution are shown in Figure 1a. It can be seen that 75% of the proteins added to the initial solution (supernatant) were adsorbed on the alginate hydrogel beads in the first 10 min. After that, the process slowed down significantly, and at the last measurement point (t = 120 min), the protein concentration in the supernatant was only 12% of the initial value. Until the end of the process, a total of 88% of proteins were adsorbed, which represents high efficiency of the process itself and confirms the suitability of alginate hydrogels as materials for microencapsulation of protein molecules. The total mass of the BSA proteins absorbed at the end of the process was 0.036 g/g beads (Figure 1d). According to the literature data, alginate is a highly charged polysaccharide and is, therefore, able to form biphasic systems with low-charged globular proteins [37], which could explain the high levels of adsorbed BSA achieved in this study. Furthermore, electrostatic interactions were also detected between other protein types and alginate hydrogels during the adsorption process: e.g., in the soy protein adsorption process, electrostatic interactions are the most important factors for determining the total adsorbed protein. The process itself is pH-dependent, with the highest rate of soy protein adsorption achieved at pH = 3.5, at which the protein is positively and alginate is negatively charged [38].

By comparing the results for the adsorption of glucose, gallic acid, and BSA, it is visible that the degree of adsorption on alginate hydrogel microcapsules was convincingly the highest for BSA (88%), followed by glucose (60%) and gallic acid (20%). In this case, the molecule with the lowest molecular weight showed the lowest adsorption properties. In general, alginate hydrogel microcapsules encapsulated with hydrophilic compounds or aqueous extracts have the characteristic of rapid release of substances, due to the porous structure of alginate hydrogels, which does not act as a barrier to such encapsulated substances [35]. Furthermore, BSA as a protein is bound to alginate gel structures by electrostatic protein–polysaccharide bonds, whereby the oppositely charged amino acids on BSA are bound to the anionic polysaccharide molecules in the alginate [39]. Due to such cross-electrostatic bonds, this complex is more stable than a mixture of alginate and smaller hydrophilic molecules, such as glucose and gallic acid, and, thus, the BSA protein exhibits higher encapsulation efficiency. 

### 2.2. Desorption (Release) Experiments

**Figure 2:**
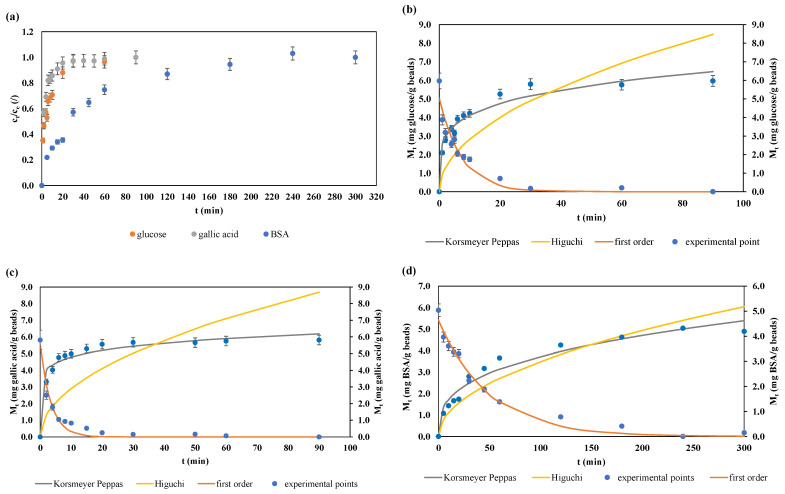
(**a**) Changes in the relative (c_t/_c_e_) concentrations of the supernatant solutions during desorption and the concentrations of the adsorbate in the alginate hydrogel beads (M_t_) during desorption (experimental + model fitted data): glucose-containing alginate hydrogel beads (**b**); gallic acid–alginate hydrogel beads (**c**) and BSA–alginate hydrogel beads (**d**). Due to model fitting procedure, data for the first-order model are shown separately (secondary axis) to the Korsmeyer–Peppas and the Higuchi model.

Changes in the relative (c_t_/c_e_) concentrations of the supernatant solutions and the mass of the released biological molecule from the alginate beads during desorption are shown in Figure 2.

The release profiles of glucose, seen as the changes in relative concentration of the solution containing the beads (Figure 2a), show a steep rise during the first five minutes of the process. The release of glucose then gradually slows down, and after 20 min it reaches a stationary state. Changes in concentration of glucose in the beads show that the initial concentration of glucose in the beads (5.967 mg/g beads) reaches almost 0 (0.177 mg/g beads) after 30 min and drops to 0 after 90 min of the process (Figure 1b). From the presented results, it is evident that the process of desorption of glucose from alginate hydrogel beads begins immediately after the immersion of the microcapsules in release medium, which can be attributed to the small dimensions of glucose molecules, which enables its rapid release.

In the case of gallic acid solution (Figure 2a), in the first minute of the process, a big jump in relative concentration from 0 to 0.569 can be seen, which represents more than half (~57%) of the total released substance during the process. After t = 20 min, the increase is quite small and the stationary state of the desorption process is reached. The same can be seen in Figure 2c—the initial concentration of gallic acid in the alginate hydrogel beads drops from 5.817 mg/g beads to 0.253 mg/g beads after the first 20 min, after which the changes in concentrations are minimal. Similar to glucose, a rapid release in the initial phase of the process can be related to the small size of the molecule being released, the loosely associated gallic acid present on the hydrogel beads surface that results in the initial rapid release, or the erosion and weakening of the bead matrix structure, due to polysaccharide hydrolysis [40,41]. Essifi and coworkers [35] studied the release kinetics of gallic acid from alginate hydrogel microcapsules of three different sizes. For all three sizes, the release profiles consisted of two phases: an initial rapid release phase, where 85% of gallic acid was released in the first 20 min; and the second phase, characterized by gradual and slower release. The authors offered two explanations for the fast phase, with the first being the presence of gallic acid molecules on the surface of the microcapsules. Another reason is that alginate hydrogel microcapsules containing a hydrophilic substance or an aqueous extract generally exhibit rapid release characteristics due to their porous structure that does not provide a sufficient barrier to these substances. Furthermore, the reduced release rate in the second phase was attributed to the release of gallic acid from the hydrogel microcapsule cores. This profile of bioactive substance release kinetics is also described by Benković et al. [21], who studied the release of bioactive compounds from plant extracts from alginate beads.

The desorption curve of BSA from alginate hydrogel microcapsules into the receiving aqueous solution (Figure 2a) shows a gradual increase in the concentration of BSA during the first 60 min of the experiment. After that, the increase in concentration slows down, and a steady state is reached after t = 240 min of the process. The initial concentration of the BSA molecule in the beads drops from initial 5.075 mg/g beads to the final value of 0.149 mg/g beads (Figure 2d). The gradual release of BSA from alginate hydrogel microcapsules can be attributed to the previously described noncovalent BSA–alginate bonds, which are electrostatic and hydrogen in nature [42]. On the other hand, release of the BSA protein from alginate hydrogels at different pH values was studied by Suksamran and coworkers, and they concluded that the release profiles in PBS buffer at a pH value of 7.4 are due to diffusion and erosion mechanisms. Similar to this study, they observed that approximately 100% of BSA was released in the PBS, at pH 7.4 over a period of 24 h [43]. In direct comparison of the release profiles of all three molecules tested in this study, it can be seen that gallic acid molecules are released the fastest, reaching the steady state after only 20 min of the process, followed by the glucose release, which reaches steady state after 30 min, and, finally, the BSA molecule, whose release is gradual, and the steady state is reached after 240 min.

### 2.3. Microscopic Characterization of the Alginate Beads

**Figure 3:**
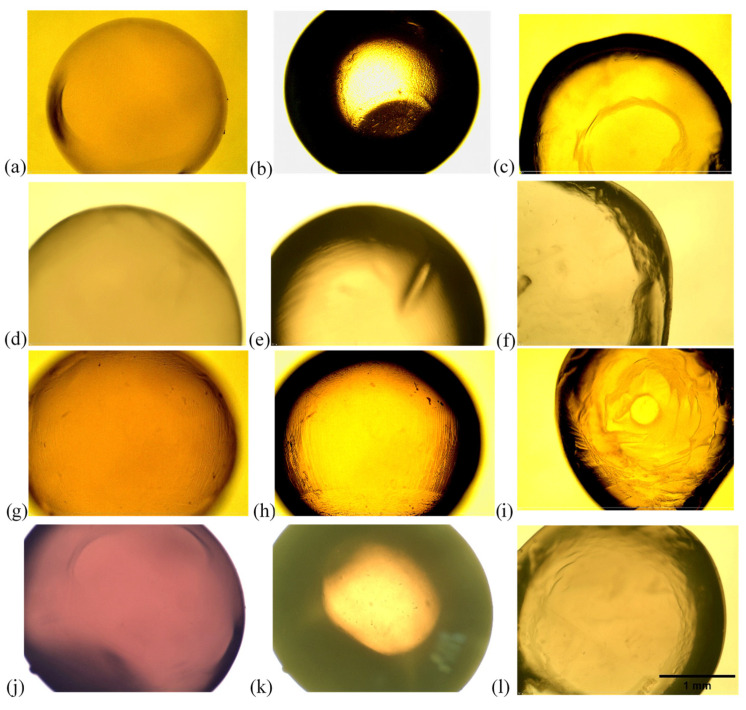
Images of the alginate beads: (**a**) plain alginate beads surface; (**b**) plain alginate beads surface with filter; (**c**) plain alginate beads cross-section; (**d**) BSA–alginate beads surface; (**e**) BSA–alginate beads surface with filter; (**f**) BSA–alginate beads cross-section; (**g**) glucose–alginate beads surface; (**h**) glucose–alginate beads surface with filter; (**i**) glucose–alginate beads cross-section; (**j**) gallic acid–alginate beads surface; (**k**) gallic acid–alginate beads surface with filter; (**l**) gallic acid–alginate beads cross-section.

The shape and morphology of hydrogel microcapsules affect their density, mechanical resistance, swelling, and their protective and release properties towards the encapsulated biocomponents [44]. Alginate hydrogel microcapsules in this research were made by the ionic gelation method, and four different types are shown in Figure 3: plain alginate, microcapsules with BSA protein, microcapsules with glucose, and microcapsules with gallic acid.

As seen in Figure 3a–l, all alginate hydrogel microcapsules are spherical, with the microcapsule without active substance having the most regular spherical shape. All microcapsules have relatively smooth and homogeneous surfaces, with the presence of rare shallow furrows on the surface of microcapsules without active substance and microcapsules with glucose. Lines and furrows are best visible in the image of a glucose containing alginate microcapsule (Figure 3h), and represent traces created when the glucose–alginate solution was squeezed out through a syringe. Folds are also visible in the image of the surface of the microcapsule with BSA (Figure 3e).

From the cross-section images (Figure 3c,f,i,l), it is evident that the wall thickness of all microcapsules is uniform in all parts. It can be observed that the outer coat is thinner on the BSA–alginate beads and gallic acid–alginate hydrogel microcapsules, while the thickest coat is visible on the glucose–alginate hydrogel microcapsule. The size of plain microcapsules shown in Figure 3a–c appears smaller compared to all those encapsulated with glucose, gallic acid, and BSA. This can be explained by the interactions between polymer chains, water molecules, glucose, gallic acid, and BSA embedded in the matrix [33].

The images obtained in this study can be compared to those of Nochos and coauthors, who prepared hydrogel microcapsules with different concentrations of alginate and a constant concentration of BSA. All microcapsules had a spherical shape, and a rough surface appearance [19]. Essifi and coworkers examined the morphology of plain alginate hydrogel microcapsules and microcapsules with gallic acid of various sizes, and their results indicated that the microcapsules had a spherical shape, with plain microcapsules having a smoother surface in comparison to those containing gallic acid [33]. 

### 2.4. Mathematical Modeling of the Adsorption and the Desorption (Release) Processes

#### 2.4.1. Adsorption Kinetics Modeling

Model-estimated parameters for the experimental data obtained during the adsorption process are shown in Table 1.

For the glucose adsorption process (Table 1), all three tested models resulted in high R^2^ values, ranging from R^2^ = 0.9321 for the Weber–Morris model to R^2^ = 0.9340 for the pseudo-first-order model and R^2^ = 0.9649 for the pseudo-second-order model, indicating that all of the models can be successfully used for a qualitative as well as quantitative description of the adsorption process. The equilibrium concentration of the adsorbed glucose determined by the pseudo-first-order and the pseudo-second-order model is fairly similar (q_e_ = 0.00042 ± 0.00025 g glucose per g beads for the pseudo-first-order and q_e_ = 0.00047 ± 0.00026 g glucose per g beads for the pseudo-second-order model), which is an indication of applicability of both models. The adsorption rate for the pseudo-first-order was 0.203 ± 0.041 min^−1^, and for the pseudo-second-order was 0.51215 ± 0.13758 g beads/g glucose per min, and the initial adsorption rates also differed (8.534 × 10^−5^ g/g beads min for the pseudo-first-order and 2.407 × 10^−4^ g/g beads min for the pseudo-second-order), which meant that the pseudo-first-order model describes the initial adsorption rate as slower compared to the pseudo-second-order model. According to Sahoo and coworkers [10], pseudo-second-order suggests that the limiting step of the process is the chemical sorption of substances, and that the adsorption rate does not depend on the adsorbate concentration, but on the capacity of the adsorbent. However, Simonin [45] emphasized that the pseudo-second-order kinetic models will often exhibit a better fit in comparison to the pseudo-first-order kinetics, due to reasons connected to the sole statistical methods used to develop the models, and that, despite high fit, the underlying mechanism behind the adsorption process does not have to be adsorption itself, but can also be diffusion in the external layer or within the particle. The Weber–Morris model, on the other hand, includes an estimation of the intraparticle diffusion rate (k_i_), as well as the diffusion resistance constant or the slope, which represents the thickness of the boundary layer (C). For glucose, the k_i_ and the C values estimated by the model indicated that the role of diffusion cannot be neglected, although its impact on the process is smaller compared to that of adsorption.

Kinetic parameters for gallic acid are also shown in Table 1. According to the obtained results (Table 1, Figure 1), there is big scattering of the experimental data, which is also seen in the model estimation: the R^2^ values were significantly lower in comparison to those obtained for glucose and BSA (R^2^ values ranged from 0.7847 for the pseudo-second-order to R^2^ = 0.4140 for the Weber–Morris model). Although they show a connection between the amount of the adsorbed substance and the duration of the process, the obtained R^2^ values are not reliable for kinetic predictions. The same can be said from the standard error values for the k_1_ and k_2_ parameters, which are markedly higher than the estimated k_1_ and k_2_ values, respectively.

For the adsorption of BSA on alginate hydrogel microcapsules, the R^2^ values for the pseudo-first and the pseudo-second-order kinetics were 0.9429 and 0.9347, respectively, while for the Weber–Morris model, the R^2^ value was 0.8167. It means that the kinetics of BSA adsorption on alginate microcapsules can be satisfactorily described by pseudo-first-order and pseudo-second-order, but pseudo-first-order is more representative. These results are in agreement with the literature data, which suggests that BSA adsorption is diffusion-based [10]. The equilibrium concentration of adsorbed BSA is 0.03299 ± 0.00269 g BSA per gram of alginate (pseudo-first-order) and 0.03795 ± 0.00409 g BSA per gram of alginate (pseudo-second-order). The adsorption rates were only significant for the pseudo-first-order (k_1_ = 0.124 ± 0.030 min^−1^ and h_0_ = 0.0041 g BSA/g beads per min), which are higher in comparison to glucose and can be explained by the electrostatic binding of BSA and alginate, as mentioned previously in the manuscript. The Weber–Morris model showed relatively high R^2^ (R^2^ = 0.8167), but only the intraparticle diffusion rate (ki) was considered significant at *p* < 0.05, and was much higher in comparison to glucose, indicating that intraparticle diffusion also plays a significant role in the process.

#### 2.4.2. Desorption (Release) Kinetics Modeling

Model-estimated parameters for the experimental data obtained during the desorption process are shown in Table 2.

To describe the desorption process of glucose, gallic acid, and BSA from the alginate hydrogel microcapsules, three kinetic models were applied—first-order kinetics, Korsmeyer–Peppas, and Higuchi. 

For the glucose desorption process (Table 2), the highest R^2^ values were demonstrated by the Korsmeyer–Peppas model (R^2^ = 0.9784), followed by the first-order-kinetic model (R^2^ = 0.9665) and the Higuchi model (R^2^ = 0.5064). The desorption rates equaled k = 0.13593 ± 0.02021 min^−1^ for the first-order, k = 2.55760 ± 0.16960 g beads/mg glucose per min) for the Korsmeyer–Peppas model, and k = 0.89343 ± 0.09836 mg/g beads min^0.5^ for the Higuchi model, respectively. The underlying mechanism of the release process was estimated by determining the value of the n parameter from the Korsmeyer–Peppas model, which had a value of n = 0.20632 ± 0.02053. This value was an indication that the release process was governed by the pseudo-Fickian diffusion mechanism [46]. In research by Lopez-Sanchez et al. [47], the n values for different glucose concentrations ranged from 0.47 to 1 for the gastric release, and from 0.49 to 0.6 for the intestinal fluid release, which led to a conclusion that the release was connected to a Fickian diffusion mechanism. However, we must emphasize that the difference in release medium also resulted in different data obtained in this study. 

Of the three models tested for the release process of the gallic acid from the alginate hydrogel microcapsules, only the first-order and the Korsmeyer–Peppas showed satisfactory results, while for the Higuchi model, no reasonable value of R^2^ was obtained due to numerical instability of the simulation. The calculated release rates were k = 0.28160 ± 0.03082 min^−1^ for the first-order kinetic model and k = 3.66236 ± 0.20776 g beads/mg min for the Korsmeyer–Peppas model. The n values were lower in comparison to those for glucose, but still in the range that shows the dominance of the pseudo-Fickian diffusion mechanism. The results are in accordance with our previous studies on the release of bioactive molecules from alginate hydrogel matrix [21].

For the description of the release process of BSA, based on the R^2^ values, all three models proved to be applicable, with the highest fit (R^2^ = 0.9899) and the lowest standard errors calculated for the first-order kinetic model. Furthermore, based on the high R^2^ values (R^2^ = 0.9794) of the Korsmeyer–Peppas model, the resulting n value proved to be significant at *p* < 0.05, and can, therefore, be reliably used as a predictor of the release mechanism. Based on those values, it can be concluded that the release mechanism of all three molecules in this study was pseudo-Fickian, meaning that it transpires in two stages—the initial fast release, followed by a slow release before reaching the equilibrium [48].

When comparing the desorption rate constants of all three tested biomolecules obtained by the Korsmeyer Peppas model, the highest values were recorded for gallic acid (k = 3.66236 ± 0.20776 g beads/mg min), followed by glucose (k = 2.55760 ± 0.16960 g beads/mg min) and BSA (k = 0.78881 ± 0.11872 g beads/mg min), thus concluding that BSA remains bound to alginate for a longer period of time due to the effect of electrostatic protein–polysaccharide bonds [49].

## 3. Conclusions

Due to its ever-growing use in microencapsulation processes in the production of food and design of drugs and cosmetic products, a comparison of the adsorption and desorption (release) kinetics of the three most commonly used biomolecules from alginate hydrogel microcapsules was conducted in this study: glucose, gallic acid as a model molecule representing polyphenols, and BSA as a model protein molecule. The experimental data were fitted to different kinetic models in order to compare the rates of adsorption and release and to determine which mechanism is the driving force of those processes. The adsorption process was best described by the pseudo-second-order kinetic model. Based on the analysis of the adsorbate concentration in the supernatant solution during the adsorption experiments, it was revealed that the highest concentration of proteins was adsorbed (88%), followed by glucose (60%) and gallic acid (20%), which is explained by the formation of electrostatic protein–polysaccharide bonds. 

By analyzing the values of the desorption rate constant (k) obtained from the Korsmeyer–Peppas model, the highest values were recorded for gallic acid, followed by glucose and BSA. According to the model-calculated values of the diffusion coefficient (n), it was determined that the desorption takes place as a two-step pseudo-Fickian diffusion process. These findings represent important insights for the design of food or pharmaceutical products, indicating that alginate hydrogel beads can be successfully used for stability enhancement of biological molecules and control of their release processes. However, further studies that include formulation optimization still need to be performed to enable further functionalization of the bioactive molecules tested in this study.

## 4. Materials and Methods

### 4.1. Materials

Sodium alginate used in this study was general-purpose grade (Fisher Scientific, Bishop Meadow Road, Loughborough, UK). The pKa values of mannuronic and guluronic acid residues of alginate are in the range 3.30–3.38 and 3.60–3.65, respectively [36,50]. The pH values of the solutions tested in this study were as follows: pH = 8.20 for glucose, pH = 3.26 for the gallic acid, pH = 7.24 for BSA, and pH = 7.45 for distilled water. Other materials and reagents were also used in the experiments: calcium chloride (GRAM-MOL, Zagreb, Croatia); bovine serum albumin (BSA) protein (Mr = 66,463 g/mol, Sigma Aldrich, St. Louis, USA); glucose (Mr = 180.156 g/mol, GRAM-MOL, Zagreb, Croatia); gallic acid 98% (Mr =170.12 g/mol, Acros Organics, Geel, Belgium); Coomasie Brilliant Blue G250 (Sigma Aldrich, St. Louis, MO, USA); Folin–Ciocalteu reagent (Kemika, Zagreb, Croatia); sodium carbonate (Kemika, Zagreb, Croatia); ethanol (J.T. Baker, Deventer, The Netherlands); glucose PAP test kit (Dijagnostika, Sisak, Croatia); phosphoric acid (85%) (Carlo Erba, Emmendingen, Germany); distilled water.

### 4.2. Methods

#### 4.2.1. Adsorption Experiments

##### Production of Plain Alginate Beads for the Adsorption Experiments

A solution of 2% (*w*/*v*) sodium alginate in distilled water was prepared using a Superior XB986F kitchen immersion blender (ZHG, Offenburg, Germany) to ensure good homogenization. After mixing, the solution was placed in an ultrasonic bath and refrigerated overnight to remove any excess bubbles left over after homogenization. The alginate solution was then transferred into a syringe, a medical needle was placed on the syringe, and the alginate solution was squeezed out manually into the 2% (*w*/*v*) CaCl_2_ receiving solution [21] in order to enable alginate gelling. After preparation, the alginate hydrogel microcapsules were left in the CaCl_2_ solution overnight to stabilize, then were filtered and thoroughly washed to remove the remains of calcium ions from the surface of the microcapsules. After washing, the capsules were stored in a refrigerator at 4–6 °C.

##### Adsorption Experiments Procedure

Before starting the adsorption experiments, 1000 plain alginate hydrogel microcapsules were separated for each experiment, and three different solutions (glucose, gallic acid, and BSA) containing the adsorbate were prepared by dissolving the adsorbate in distilled water. Solution concentrations for different adsorbates were 100 mg/L for the glucose solution (pH = 8.20), 5 g/L for the gallic acid solution (pH = 3.26), and 0.5% (*w*/*v*) BSA solution (pH = 7.24). The solutions were then placed in a heated magnetic oil bath (IKA HBR 4 digital, IKA-Werk, Staufen, Germany) at 30 °C and 200 rpm, and 1000 plain beads were placed in each adsorbate solution [51]. Immediately after the addition of the alginate hydrogel beads, a stop clock was started and the supernatant samples were taken in the following time intervals: for the glucose solution, t = 0, 1, 2, 3, 4, 5, 10, 20, 30, 40, 50, 60, and 90 min; for the gallic acid solution, t = 0, 2, 4, 6, 8, 10, 20, 30, 40, 50, 60, 90, 120, and 180 min; and for the BSA solution, t = 0, 1, 2, 3, 4, 5, 6, 8, 10, 20, 30, 60, and 120 min. Samples were placed in Eppendorf tubes and stored in a freezer until further analysis.

#### 4.2.2. Desorption Experiments

##### Production of Adsorbate-Containing Alginate Beads for the Desorption Experiments

Alginate hydrogel beads intended for the desorption experiments were produced in the same manner as the plain alginate hydrogel beads, with one exception—the alginate powder (2% *w*/*v*) was dissolved in the adsorbate containing solutions instead of distilled water. The adsorbate solution concentrations mixed with alginate powder were glucose 0.5% *w*/*v*, gallic acid 0.5% *w*/*v,* and BSA 0.5% *w*/*v*. After preparation of hydrogel microbeads by extrusion dripping, the microcapsules were left in their corresponding solutions (2% CaCl_2_ solution in glucose, gallic acid, or BSA) overnight to stabilize, and then were filtered and thoroughly washed to remove the remains of calcium ions from the surface of the microcapsules. After washing, the capsules were stored in a refrigerator at 4–6 °C.

##### Desorption Experiments Procedure

Desorption experiments were conducted for each biological molecule separately. An amount of 1000 hydrogel beads were separated and placed in a glass containing distilled water (pH = 7.45) in an oil bath at 30 °C and 200 rpm [51]. For each biological molecule, samples of the supernatants were taken in the following intervals: for glucose, t = 0, 1, 2, 3, 4, 5, 6, 8, 10, 20, 30, 40, 50, 60, and 90 min; for gallic acid, t = 0, 2, 4, 6, 8, 10, 15, 20, 30, 40, 50, 60, and 90 min; for BSA, t = 0, 5, 10, 15, 20, 30, 45, 60, 120, 180, 240, and 300 min. Samples were placed in Eppendorf tubes and stored in a freezer until further analysis.

#### 4.2.3. Microscopic Characterization of the Alginate Hydrogel Microbeads

All types of prepared microcapsules (plain, microbeads after adsorption, and microbeads after desorption) were recorded using a Motic B series light microscope with a Moticam 3 camera (Moticam, Barcelona, Spain) under 4× magnification. The surface appearance and cross-section of all microcapsule samples were analyzed.

#### 4.2.4. Analysis of the Supernatants Collected during the Adsorption and Desorption Experiments

##### Glucose Concentration Measurement

To measure the glucose concentration, a PAP reagent was used (glucose oxidase, >15,000 U/L; peroxidase >100 U/L; 4-aminoantipyrine 0.3 mmol/L; phenol 0.5 mmol/L; and phosphate buffer pH 7.1 ± 0.2), which was prepared by dissolving the contents of the vial supplied in the test kit in 250 mL of distilled water. After measuring the absorbance of the empty cuvettes, 1 mL of PAP solution and 10 µL of the sample were added directly to the cuvettes, vortexed, and the absorbance was measured at 500 nm after a 30 min incubation at room temperature. Glucose concentration was then calculated based on the calibration curve. Measurements were performed in triplicate and the results were expressed as g/L. 

##### Gallic Acid Concentration Measurement Using the Folin–Ciocalteu Reagent

The concentration of gallic acid as a model polyphenol molecule was determined spectrophotometrically using the Folin–Ciocalteu reagent, as described previously by Singleton and Rossi [52]. Briefly, 7.9 mL of distilled water, 100 μL of sample, 500 μL of Folin–Ciocalteu reagent, and 1.5 mL of 20% Na_2_CO_3_ solution were pipetted into the test tube. The samples were then incubated at room temperature in the dark for 2 h, and the absorbance of the developed blue color was measured spectrophotometrically at 765 nm. The unknown concentration of gallic acid was then calculated from the calibration curve made with known gallic acid concentrations (0, 25, 50, 75, 100, 200, 250, 400, and 500 mg/L). Measurements were performed in triplicate and the results were expressed as mg GAE/L. 

##### Protein Concentration Measurement

Protein concentration in samples was determined spectrophotometrically (λ = 595 nm) according to the literature [53] with some modifications. Briefly, Bradford’s reagent was prepared by mixing 100 mg of Coomassie Brilliant Blue G250 reagent with 50 mL of ethanol, 100 mL of H_3_PO_4_ (85%), and 850 mL of distilled water. After measuring the absorbance of plastic cuvettes with distilled water at λ = 595 nm (blank), a reaction mixture containing 500 µL of Bradford’s reagent and 500 µL of the sample was vortexed and incubated for 10 min at room temperature. The absorbance was measured at 595 nm and the unknown protein concentration was then calculated based on the calibration curve of known protein concentrations (0, 1, 5, 10, 15, and 20 mg/L). Measurements were performed in triplicate and the results were expressed as g/L.

#### 4.2.5. Data Analysis and Mathematical Modeling

Data analysis and mathematical modeling for the adsorption and desorption processes of biological molecules from alginate hydrogel microbeads were performed using the Statistica v. 14.0. software package (TibcoStatistica, Palo Alto, Santa Clara, CA, USA), with a probability level *p* < 0.05. To analyze the adsorption kinetics, the amount of adsorbate adsorbed on the hydrogel beads was calculated according to Equation (1):

(1) $$q_{t}=\frac{\left(c_{0}-c_{t}\right)\cdot V}{m}$$

where q_t_ is the amount of adsorbed solute (adsorbate) at time t (g/g beads), c_0_ is the concentration of the solution at time t = 0 (g/mL), c_t_ is the concentration of the solution at time t (g/mL), V is the volume of the adsorbate solution (L), and m is the mass of the adsorbents (beads; g).

Experimental data derived from the adsorption experiments were fitted to three different models: pseudo-first-order (Equation (2)), pseudo-second-order (Equation (3)), and the Weber–Morris interparticle diffusion model (Equation (4)):

(2) $$q_{t}=q_{e}\left[1-exp({-k}_{1}t)\right];h_{0}=k_{1}q_{e}$$

(3) $$q_{t}=\frac{k_{2}q_{e}^{2}t}{1+k_{2}q_{e}t};\;h_{0}=k_{2}q_{e}^{2}$$

(4) $$q_{t}=k_{i}t^{0.5}+C$$

where q_t_ represents the concentration of the adsorbed adsorbate (glucose, gallic acid, or BSA)(g/g) in time t, q_e_ represents the equilibrium concentration, i.e., adsorption capacity (g/g); k_1_ is the adsorption rate for pseudo-first-order kinetics (min^−1^); k_2_ is the adsorption rate for pseudo-second-order kinetics (g/g min); h_0_ is the initial adsorption rate (g/g min); ki is the intraparticle diffusion rate (g/g min^0.5^); and C is the diffusion resistance constant or the slope which represents the thickness of the boundary layer (g/g) [54].

For estimation of kinetic parameters for desorption, the amount of adsorbate desorbed from the alginate hydrogel beads was calculated according to Equation (5):

(5) $$M_{t}=\frac{\left(C_{e}-C_{t}\right)V}{m}$$

where M_t_ is the amount of desorbed glucose, gallic acid, or BSA at time t (mg/g beads), C_e_ is the final concentration of the solution at time t = 0 (g/mL), C_t_ is the concentration of the solution at time t (g/mL), V is the volume of the solution used for desorption (L), and m is the mass of the beads used for desorption (g).

For the desorption experiments, data obtained from the experiments were fitted to the first-order (Equation (6)), Korsmeyer–Peppas (Equation (7)), and the Higuchi model (Equation (8)):

(6) $$M_{t}=M_{e}e^{-kt}$$

(7) $$M_{t}={kt}^{n}$$

(8) $$M_{t}={kt}^{0.5}$$

where M_t_ (mg/g) represents the amount of released adsorbate in solution in time t (min), M_e_ is the final concentration of the adsorbate in the solution (mg/g); k is the desorption rate (min^−1^); and n is the diffusion coefficient. Values of the parameter n define the dominant process during desorption of adsorbate from hydrogel adsorbents: n < 0.5 means pseudo-Fickian diffusion; n = 0.5 means Fickian diffusion; 0.5 < n < 1 means hybrid diffusion mechanism; and n = 1 means diffusion that does not follow the Fick’s law [46,51]. The adequacy of the proposed models was estimated based on the coefficient of determination (R^2^) values and the standard error (SE) values for each model-estimated parameter.

#### 4.2.6. Statistical Analysis

Basic statistical analysis (mean and standard error values) of three parallel replicates for each analysis performed in this study was calculated using the Statistica v. 14.0. software (TibcoStatistica, Palo Alto, USA). Furthermore, all the models were developed using all three parallel values obtained from the experiments, which equals (in total) 39 data points for glucose adsorption modeling, 42 data points for gallic acid adsorption modeling, 39 data points for BSA adsorption modeling, 45 data points for glucose desorption modeling, 39 data points for gallic acid desorption modeling, and 36 data points for the BSA desorption modeling. Models were developed using the same software package (Statistica v. 14.0), with an implementation of the Levenberg–Marquardt algorithm and a probability level *p* < 0.05.

## Figures and Tables

**Table 1:** Model-estimated parameters of the adsorption experiments. Values are expressed as mean ± standard error. Model-obtained values presented in bold are considered significant at *p* < 0.05.

Model/Parameter	Glucose	Gallic Acid	BSA
Pseudo-first-order			
q_e_ (g/g beads)	**0.00042 ± 0.00025**	**0.00855 ± 0.00612**	**0.03299 ± 0.00269**
k_1_ (min^−1^)	**0.203 ± 0.041**	2.008 ± 7.12471	**0.124 ± 0.030**
h_0_ (g/g beads min)	**8.534 × 10^−5^**	0.017	**0.0041**
R^2^	0.9340	0.7469	0.9429
Pseudo-second-order			
q_e_ (g/g beads)	**0.00047 ± 0.00026**	**0.0087 ± 0.0010**	**0.03795 ± 0.00409**
k_2_ (g beads/g min)	**0.51215 ± 0.13758**	771.0248 ± 2914.105	3.6776 ± 1.6348
h_0_ (g/g beads min)	**2.407 × 10^−4^**	0.0583	0.00530
R^2^	0.9649	0.7847	0.9347
Weber–Morris			
k_i_ (g/g beads min^0.5^)	**0.000047 ± 0.0000**	0.000324 ± 0.000205	**0.00338 ± 0.00080**
C (g/g)	**0.00016 ± 0.00003**	**0.00615 ± 0.001137**	0.006536 ± 0.00388
R^2^	0.9321	0.4140	0.8167

**Table 2:** Model-estimated parameters of the desorption experiments. Values are expressed as mean ± standard error. Model-obtained values presented bold are considered significant at *p* < 0.05.

Model/Parameter	Glucose	Gallic Acid	BSA
First-order			
M_e_ (mg/g beads)	**5.0368 ± 0.34470**	**5.52911 ± 0.32344**	**4.64360 ± 0.16520**
k (min^−1^)	**0.13593 ± 0.02021**	**0.28160 ± 0.03082**	**0.02019 ± 0.00188**
R^2^	0.9665	0.9804	0.9899
Korsmeyer–Peppas			
k (g beads/mg min)	**2.55760 ± 0.16960**	**3.66236 ± 0.20776**	**0.78881 ± 0.11872**
n	**0.20632 ± 0.02053**	**0.11653 ± 0.01767**	**0.33692 ± 0.03077**
R^2^	0.9784	0.9813	0.9794
Higuchi			
k (mg/g beads min^0.5^)	**0.89343 ± 0.09836**	0.91613 ± 0.11281	**0.34863 ± 0.01965**
R^2^	0.5064	/	0.9264

## Data Availability

Data are contained within the article.

## References

[1] Corrêa-Filho L.C., Moldão-Martins M., Alves V.D. (2019). Advances in the Application of Microcapsules as Carriers of Functional Compounds for Food Products. Appl. Sci..

[2] Puscaselu R.G., Lobiuc A., Dimian M., Covasa M. (2020). Alginate: From Food Industry to Biomedical Applications and Management of Metabolic Disorders. Polymers.

[3] Brownlee I.A., Seal C.J., Wilcox M., Dettmar P.W., Pearson J.P. (2009). Applications of Alginates in Food. Alginates Biol. Appl..

[4] Bi D., Yang X., Yao L., Hu Z., Li H., Xu X., Lu J. (2022). Potential Food and Nutraceutical Applications of Alginate: A Review. Mar. Drugs.

[5] Yim Z.H., Tiong C.B., Mansa R.F., Ravindra P., Chan E.S. (2010). Release Kinetics of Encapsulated Herbal Antioxidants during Gelation Process. J. Appl. Sci..

[6] Yudaev P., Semenova A., Chistyakov E. (2024). Gel Based on Modified Chitosan for Oil Spill Cleanup. J. Appl. Polym. Sci..

[7] Peng X., Umer M., Pervez M.N., Hasan K.M.F., Habib M.A., Islam M.S., Lin L., Xiong X., Naddeo V., Cai Y. (2023). Biopolymers-Based Microencapsulation Technology for Sustainable Textiles Development: A Short Review. Case Stud. Chem. Environ. Eng..

[8] Plazinski W., Dziuba J., Rudzinski W. (2013). Modeling of Sorption Kinetics: The Pseudo-Second Order Equation and the Sorbate Intraparticle Diffusivity. Adsorption.

[9] Unuabonah E.I., Omorogie M.O., Oladoja N.A. (2019). Modeling in Adsorption: Fundamentals and Applications. Composite Nanoadsorbents.

[10] Sahoo T.R., Prelot B. (2020). Adsorption Processes for the Removal of Contaminants from Wastewater: The Perspective Role of Nanomaterials and Nanotechnology. Nanomaterials for the Detection and Removal of Wastewater Pollutants.

[11] Sharma G., Naushad M., Kumar A., Rana S., Sharma S., Bhatnagar A., Stadler F.J., Ghfar A.A., Khan M.R. (2017). Efficient Removal of Coomassie Brilliant Blue R-250 Dye Using Starch/Poly(Alginic Acid-Cl-Acrylamide) Nanohydrogel. Process Saf. Environ. Prot..

[12] Bruschi M.L. (2015). Strategies to Modify the Drug Release from Pharmaceutical Systems.

[13] Seida Y., Tokuyama H. (2022). Hydrogel Adsorbents for the Removal of Hazardous Pollutants—Requirements and Available Functions as Adsorbent. Gels.

[14] Hossain M.S., Hossain M.M., Khatun M.K., Hossain K.R. (2024). Hydrogel-Based Superadsorbents for Efficient Removal of Heavy Metals in Industrial Wastewater Treatment and Environmental Conservation. Environ. Funct. Mater..

[15] Darban Z., Shahabuddin S., Gaur R., Ahmad I., Sridewi N. (2022). Hydrogel-Based Adsorbent Material for the Effective Removal of Heavy Metals from Wastewater: A Comprehensive Review. Gels.

[16] Zieman J., Cohan M., Wang Y., De La Sancha A., Kanungo M., Azzouz R., Smith R., Schmidt K., Kumpaty S., Chen J. (2023). Development of Gelatin-Coated Hydrogel Microspheres for Novel Bioink Design: A Crosslinker Study. Pharmaceutics.

[17] Motelica L., Ficai D., Oprea O.C., Ficai A., Ene V.L., Vasile B.S., Andronescu E., Holban A.M. (2021). Antibacterial Biodegradable Films Based on Alginate with Silver Nanoparticles and Lemongrass Essential Oil–Innovative Packaging for Cheese. Nanomaterials.

[18] Geetha P., Latha M.S., Koshy M. (2015). Biosorption of Malachite Green Dye from Aqueous Solution by Calcium Alginate Nanoparticles: Equilibrium Study. J. Mol. Liq..

[19] Nochos A., Douroumis D., Bouropoulos N. (2008). In Vitro Release of Bovine Serum Albumin from Alginate/HPMC Hydrogel Beads. Carbohydr. Polym..

[20] Bušić A., Belščak-Cvitanović A., Vojvodić Cebin A., Karlović S., Kovač V., Špoljarić I., Mršić G., Komes D. (2018). Structuring New Alginate Network Aimed for Delivery of Dandelion (*Taraxacum officinale* L.) Polyphenols Using Ionic Gelation and New Filler Materials. Food Res. Int..

[21] Benković M., Sarić I., Jurinjak Tušek A., Jurina T., Gajdoš Kljusurić J., Valinger D. (2021). Analysis of the Adsorption and Release Processes of Bioactives from Lamiaceae Plant Extracts on Alginate Microbeads. Food Bioprocess Technol..

[22] Lopez-Sanchez P., Assifaoui A., Cousin F., Moser J., Bonilla M.R., Ström A. (2022). Impact of Glucose on the Nanostructure and Mechanical Properties of Calcium-Alginate Hydrogels. Gels.

[23] Li W., Chen W., Wang Z., Chen W., Zhang M., Zhong Q., Chen H., Pei J. (2022). Preparation and Characterization of Beads of Sodium Alginate/Carboxymethyl Chitosan/Cellulose Nanofiber Containing Porous Starch Embedded with Gallic Acid: An In Vitro Simulation Delivery Study. Foods.

[24] Yamagiwa K., Kozawa T., Ohkawa A. (1995). Effects of Alginate Composition and Gelling Conditions on Diffusional and Mechanical Properties of Calcium-Alginate Gel Beads. J. Chem. Eng. Jpn..

[25] Jiao W., Chen W., Mei Y., Yun Y., Wang B., Zhong Q., Chen H., Chen W. (2019). Effects of Molecular Weight and Guluronic Acid/Mannuronic Acid Ratio on the Rheological Behavior and Stabilizing Property of Sodium Alginate. Molecules.

[26] Mohammed C., Lalgee L., Kistow M., Jalsa N., Ward K. (2022). On the Binding Affinity and Thermodynamics of Sodium Alginate-Heavy Metal Ion Interactions for Efficient Adsorption. Carbohydr. Polym. Technol. Appl..

[27] Ramos P.E., Silva P., Alario M.M., Pastrana L.M., Teixeira J.A., Cerqueira M.A., Vicente A.A. (2018). Effect of Alginate Molecular Weight and M/G Ratio in Beads Properties Foreseeing the Protection of Probiotics. Food Hydrocoll..

[28] Tanaka H., Matsumura M., Veliky I.A. (1984). Diffusion Characteristics of Substrates in Ca-Alginate Gel Beads. Biotechnol. Bioeng..

[29] Mehmetoğlu Ü., Hacimusalar M. (1992). Determination of the Effective Diffusion Coefficient of Glucose in Calcium Alginate Gel Using the Moment Analysis Technique. Recent Advances in Biotechnology.

[30] Venâncio A., Teixeira J.A. (1997). Characterization of Sugar Diffusion Coefficients in Alginate Membranes. Biotechnol. Tech..

[31] Kuo C.K., Ma P.X. (2001). Controlling Diffusion of Solutes through Ionically Crosslinked Alginate Hydrogels Designed for Tissue Engineering. Mater. Res. Soc. Symp.-Proc..

[32] Kalam S., Abu-Khamsin S.A., Kamal M.S., Patil S. (2021). Surfactant Adsorption Isotherms: A Review. ACS Omega.

[33] Essifi K., Brahmi M., Berraaouan D., Ed-Daoui A., El Bachiri A., Fauconnier M.L., Tahani A. (2021). Influence of Sodium Alginate Concentration on Microcapsules Properties Foreseeing the Protection and Controlled Release of Bioactive Substances. J. Chem..

[34] Machado A.R., Silva P.M.P., Vicente A.A., Souza-Soares L.A., Pinheiro A.C., Cerqueira M.A. (2022). Alginate Particles for Encapsulation of Phenolic Extract from Spirulina Sp. LEB-18: Physicochemical Characterization and Assessment of In Vitro Gastrointestinal Behavior. Polymers.

[35] Essifi K., Lakrat M., Berraaouan D., Fauconnier M.L., El Bachiri A., Tahani A. (2021). Optimization of Gallic Acid Encapsulation in Calcium Alginate Microbeads Using Box-Behnken Experimental Design. Polym. Bull..

[36] Chuang J.J., Huang Y.Y., Lo S.H., Hsu T.F., Huang W.Y., Huang S.L., Lin Y.S. (2017). Effects of PH on the Shape of Alginate Particles and Its Release Behavior. Int. J. Polym. Sci..

[37] Michaux M., Salinas N., Miras J., Vílchez S., González-Azón C., Esquena J. (2021). Encapsulation of BSA/Alginate Water–in–Water Emulsions by Polyelectrolyte Complexation. Food Hydrocoll..

[38] Silverio G.B., Sakanaka L.S., Alvim I.D., Shirai M.A., Grosso C.R.F. (2018). Production and Characterization of Alginate Microparticles Obtained by Ionic Gelation and Electrostatic Adsorption of Concentrated Soy Protein. Ciência Rural.

[39] Zhao Y., Li F., Carvajal M.T., Harris M.T. (2009). Interactions between Bovine Serum Albumin and Alginate: An Evaluation of Alginate as Protein Carrier. J. Colloid Interface Sci..

[40] Belščak-Cvitanovic A., Bušić A., Barišić L., Vrsaljko D., Karlović S., Špoljarić I., Vojvodić A., Mršić G., Komes D. (2016). Emulsion Templated Microencapsulation of Dandelion (*Taraxacum officinale* L.) Polyphenols and β-Carotene by Ionotropic Gelation of Alginate and Pectin. Food Hydrocoll..

[41] Lozano-Vazquez G., Lobato-Calleros C., Escalona-Buendia H., Chavez G., Alvarez-Ramirez J., Vernon-Carter E.J. (2015). Effect of the Weight Ratio of Alginate-Modified Tapioca Starch on the Physicochemical Properties and Release Kinetics of Chlorogenic Acid Containing Beads. Food Hydrocoll..

[42] Xu X., Han Q., Shi J., Zhang H., Wang Y. (2020). Structural, Thermal and Rheological Characterization of Bovine Serum Albumin Binding with Sodium Alginate. J. Mol. Liq..

[43] Suksamran T., Opanasopit P., Rojanarata T., Ngawhirunpat T., Ruktanonchai U., Supaphol P. (2009). Biodegradable Alginate Microparticles Developed by Electrohydrodynamic Spraying Techniques for Oral Delivery of Protein. J. Microencapsul..

[44] Dima C., Pətraşcu L., Cantaragiu A., Alexe P., Dima Ş. (2016). The Kinetics of the Swelling Process and the Release Mechanisms of *Coriandrum sativum* L. Essential Oil from Chitosan/Alginate/Inulin Microcapsules. Food Chem..

[45] Simonin J.P. (2016). On the Comparison of Pseudo-First Order and Pseudo-Second Order Rate Laws in the Modeling of Adsorption Kinetics. Chem. Eng. J..

[46] Rezaei A., Nasirpour A. (2019). Evaluation of Release Kinetics and Mechanisms of Curcumin and Curcumin-β-Cyclodextrin Inclusion Complex Incorporated in Electrospun Almond Gum/PVA Nanofibers in Simulated Saliva and Simulated Gastrointestinal Conditions. Bionanoscience.

[47] Lopez-Sanchez P., Fredriksson N., Larsson A., Altskär A., Ström A. (2018). High Sugar Content Impacts Microstructure, Mechanics and Release of Calcium-Alginate Gels. Food Hydrocoll..

[48] Wind M.M., Lenderink H.J.W. (1996). A Capacitance Study of Pseudo-Fickian Diffusion in Glassy Polymer Coatings. Prog. Org. Coat..

[49] Neiser S., Draget K.I., Smidsrød O. (1999). Interactions in Bovine Serum Albumin–Calcium Alginate Gel Systems. Food Hydrocoll..

[50] Sreya E.S., Kumar D.P., Balakrishnan P., Gopi S. (2023). Science and Technology of Alginates: A Review. Handbook of Biomass.

[51] Birkić A., Valinger D., Jurinjak Tušek A., Jurina T., Gajdoš Kljusurić J., Benković M. (2021). Evaluation of the Adsorption and Desorption Dynamics of Beet Juice Red Dye on Alginate Microbeads. Gels.

[52] Singleton V.L., Rossi J.A. (1965). Colorimetry of Total Phenolics with Phosphomolybdic-Phosphotungstic Acid Reagents. Am. J. Enol. Vitic..

[53] Kielkopf C.L., Bauer W., Urbatsch I.L. (2020). Bradford Assay for Determining Protein Concentration. Cold Spring Harb. Protoc..

[54] Dahri M.K., Kooh M.R.R., Lim L.B.L. (2015). Application of Casuarina Equisetifolia Needle for the Removal of Methylene Blue and Malachite Green Dyes from Aqueous Solution. Alex. Eng. J..

